# Kinematic Measurements of Novel Chaotic Micromixers to Enhance Mixing Performances at Low Reynolds Numbers: Comparative Study

**DOI:** 10.3390/mi12040364

**Published:** 2021-03-28

**Authors:** Toufik Tayeb Naas, Shakhawat Hossain, Muhammad Aslam, Arifur Rahman, A. S. M. Hoque, Kwang-Yong Kim, S. M. Riazul Islam

**Affiliations:** 1 Gas Turbine Joint Research Team, University of Djelfa, 17000 Djelfa, Algeria; toufiknaas@gmail.com; 2 Department of Industrial and Production Engineering, Jashore University of Science and Technology, Jashore 7408, Bangladesh; shakhawat.ipe@just.edu.bd (S.H.); asm.hoque2@mail.dcu.ie (A.S.M.H.); 3 Department of Chemical Engineering, COMSATS University Islamabad (CUI), Lahore Campus, Defense Road, Off Raiwind Road, Lahore 45550, Pakistan; maslam@cuilahore.edu.pk; 4 Department of Mechanical Engineering, Bangabandhu Textile Engineering College, Kalihati, Tangail 1970, Bangladesh; arif02me@gmail.com; 5 Department of Mechanical Engineering, Inha University, 100 Inha-Ro, Michuhol-Gu, Incheon 22212, Korea; 6 Department of Computer Science and Engineering, Sejong University, Seoul 05006, Korea

**Keywords:** TLCCM configuration, mixing rate, kinematics, deformation, vorticity, stretching, folding

## Abstract

In this work, a comparative investigation of chaotic flow behavior inside multi-layer crossing channels was numerically carried out to select suitable micromixers. New micromixers were proposed and compared with an efficient passive mixer called a Two-Layer Crossing Channel Micromixer (TLCCM), which was investigated recently. The computational evaluation was a concern to the mixing enhancement and kinematic measurements, such as vorticity, deformation, stretching, and folding rates for various low Reynolds number regimes. The 3D continuity, momentum, and species transport equations were solved by a Fluent ANSYS CFD code. For various cases of fluid regimes (0.1 to 25 values of Reynolds number), the new configuration displayed a mixing enhancement of 40%–60% relative to that obtained in the older TLCCM in terms of kinematic measurement, which was studied recently. The results revealed that all proposed micromixers have a strong secondary flow, which significantly enhances the fluid kinematic performances at low Reynolds numbers. The visualization of mass fraction and path-lines presents that the TLCCM configuration is inefficient at low Reynolds numbers, while the new designs exhibit rapid mixing with lower pressure losses. Thus, it can be used to enhance the homogenization in several microfluidic systems.

## 1. Introduction

Mass transfer induced by different fluid concentrations is generally applied in kinematic processes that involve the dynamic transport of chemical species within physical technology. The mixing fluids process of micromixers occurs in multiple applications of different mechanisms and industrial applications that have much utility in bioengineering fields, biomedical treatments, and chemical reactions [1,2,3], etc.

The role of mass transfer is a subject of interest for many researchers who have given solutions to enhance the kinematic behavior and fluid mixing performance while reducing pressure drops. However, due to the small size of micromixers, it is difficult to enhance the molecular distribution operation during fluid flow, while the improvement in dynamics and kinematic behaviors is limited. The generation of secondary flows through chaotic advection is a suitable technique to enhance mixing efficiency using passive micromixers [4,5].

To understand the behavior of mass fraction inside T-type micromixers, many researchers have tried to analyze solutions within split and recombined micromixers [6] or modified curved T-type mixers [4]. The impacts of various flow regimes on velocity contours, initial sensitivity, mixing capacity, pressure drop, energy performances, and entropy generation rates were investigated numerically and experimentally. Their results showed that for low flow regimes, the molecular diffusion dominated for mixing fluid; therefore, a high mixing performance occurred for all configurations. In addition, the strong secondary flows in the proposed configuration enhanced the entropy generation. In real-time, Huanhuan et al. [7] investigated fluid mixing via a serpentine micromixer based on an elliptical groove design. The results indicated a significantly enhanced mixing performance (90%). Numerical works were performed to characterize the dynamic flow of Newtonian fluids for complex micromixers, such as zigzag, square-wave [8], and two-layer crossing channel [9,10]. The authors argued that the best mixing rate was obtained for a chaotic configuration with low cost. For low Reynolds numbers, Koudari et al. [11] investigated the quality of mixing flow of Newtonian fluids within a two-layer micromixer, which was proposed recently by Shakhawat et al. [12]. The authors modified the crossing zone to reduce the number of units and enhance the mixing performance through a parametric study. The authors also solved the species transport equations using CFD software. Arshad and Kim [10] found that a convergent-divergent micromixer with a T-junction described the most efficient coupling with pulsatile flow. The mass transfer showed that the interfacial area rises and separates to produce discrete puffs of flows for enhanced mixing efficiency. Chih et al. [13] proposed a new procedure to enhance fluid mixing in T-shaped micromixers by adding vortex-inducing obstacles. They present the particle-tracking flow with an estimation of diffusion prototypes to determine the mass distribution within the micromixer. An experimental study was carried out to fabricate the fused silica microfluidic mixer via employing low viscosity, by Dena et al. [14]. They illustrated the utilization of a newly fabricated mixer to examine the folding of the Pin1 WW region at a low time range.

Recently, a new technique to create a strong vortex inside the fluid flow by using a direct current (DC) electric field was investigated [15,16]. Newtonian fluid flow in T-channel [15] and corrugated wall [16] configurations were suggested to improve the mixing process. They found that the fluid homogenization rate enhances more than 200% by adding a direct current (DC) electric field inside the configurations. To characterize mixing efficiency with secondary flows, researchers use a different parameter focused on both fluid homogenization and fluid kinematic behavior for active [17] and passive mixers [18,19,20] called Local Physical Process (LPP) or kinematic measurements. They analyzed the behavior of the fluid in motion by calculating elongation, vorticity, helicity, stretching, and folding rates for different flow regimes.

In this work, novel multi-layer micromixers have been proposed to offer excellent kinematic performances and mixing enhancement with low-pressure drops, compared with a recent two-layer micromixer created by Kouadri et al. [11]. Kinematic measurements of new chaotic micromixers at low Reynolds numbers are investigated in detail.

## 2. Geometries Description and Boundary Conditions

**Figure 1:**
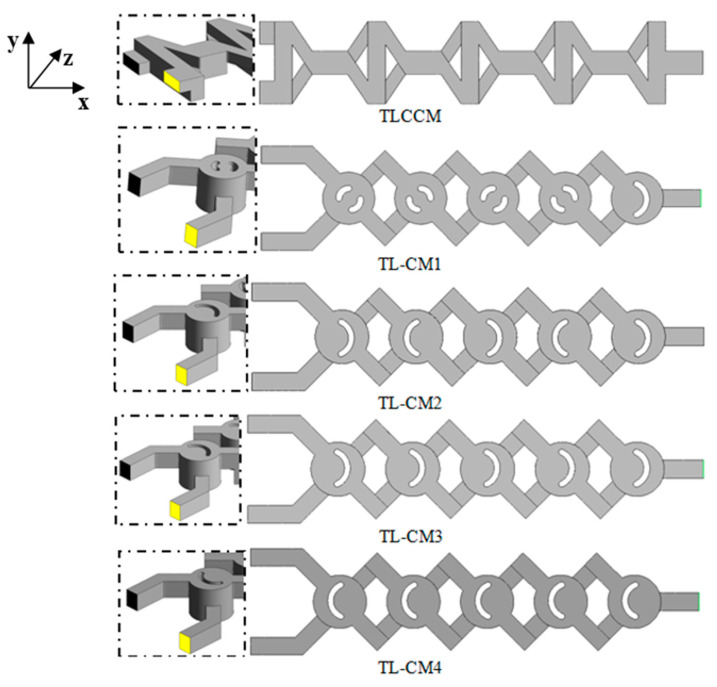
Schematic representation of the micromixers with geometric parameters.

New chaotic multi-layer micromixers alongside the relevant boundary conditions are proposed, namely, Two-Layer Crossing Modified geometry (TLC-M1, TLC-M2, TLC-M3, and TLC-M4) compared with the Two-Layer Crossing Channel Micromixer (TLCCM), which was used recently by Kouadri et al. [11] and Shakhawat et al. [12], in terms of kinematic behavior and mixing process. This micromixer is formed of two twisted channels of the same dimensions; the upper and lower channels are arranged with a periodic chamber. The serial mixing parts occur in reconstructed models of various grooves. Each mixer is created with an unfolded length equal to 7.5 mm, the same width and depth of 1.5 mm, and a hydraulic diameter of 1.5 mm. Figure 1 displays a 3D view design of the proposed micromixers. Two water liquids are imposed at the inlet sections with different colors and uniform velocity. The outsides are supposed to be adiabatic, and other flow boundaries include no-slip. The pressure outlet condition is considered at the outflow section.

## 3. Governing Equations and Numerical Methodology

Continuity, momentum, and species transport equations of incompressible steady flows are presented by the following equations:

- Continuity equation:
  (1) $$\nabla \;\vec{V}=0$$
- Momentum equation:
  (2) $$\left(\vec{V}\nabla \right)\vec{V}=\;-\;\frac{1}{\rho }\;\nabla \;P+\;\nu \nabla^{2}\vec{V}$$
  where *P*, *V*, and *ρ* represent the static pressure, velocity, and density of the fluid, respectively. For Newtonian fluid flow, the species transport equation is expressed as follows:
  (3) $$\vec{V}\nabla =D\nabla^{2}C_{i}$$

Uniform velocities were imposed at the inlets with a no-slip boundary condition applied on the other parts of the walls, and zero static pressure at the outlet section. Additionally, in one of the fluid entries, the mass fraction equals 1, and the other equals zero.

All governing equations in the proposed micromixers were solved in a laminar regime by using ANSYS Fluent^TM^ 16 (Ansys, Canonsburg, PL, USA) CFD software [21], which is essentially based on the finite volumes method. The SIMPLEC scheme was chosen for velocity coupling and pressure. A second-order upwind scheme was selected to solve the concentration and momentum equations. The computations were ensured and simulated to be converged at 10^−7^ of root mean square (RMS) residual values. Pure water liquids were used as a working fluid, with a fluid density of 1000 kg/m^3^ and the diffusion coefficient equal to 1 × 10^−11^ m^2^/s.

The Reynolds number is defined as follows:

(4) Re= ρvDhμ

## 4. Mixing Rate

The mixing rate ($M_{r}$) of two fluids, given in the following equation, is an efficient parameter for quantifying scalar mixing:

(5) $$M_{r}=1-\frac{\sqrt{\frac{1}{N}\sum_{i=1}^{N}{\left(C_{i}-\bar{C}\right)}^{2}}}{\sigma_{0}}$$

where *N* is the number of nodes on one section, $C_{i}\;$is the mass fraction at the sampling point *i*, and $\bar{C}$ is the most favorable of the mass fraction of $C_{i}$. $\sigma_{0}$ is the standard deviation at the inlet flow section. The values of $M_{r}$ range from zero for the no mixture case, to 1 for optimum mixing.

## 5. Kinematic Behavior Process of the Chaotic Flows

The velocity field is strongly dependent on the components of the velocity gradient (∂*Ui*)/(∂*xj*). Therefore, these components contribute to the kinematic flow of the fluid, such as vortex velocity, strain rate, rotation speed, and vortex stretch and compression [18]. It should be noted that these characteristics are almost negligible for a very slow regime (Re < 1), as the fluid flow is laminar and settling.

### 5.1. Stretching and Folding Process

The transport equation of the vorticity is written and mathematically articulated as [19,20]:

(6) $$\partial \vec{\Omega }/\partial t+\vec{V}.\bar{\overset{¯}{\nabla }}\vec{\Omega }=\vec{\Omega }.\bar{\overset{¯}{\nabla }}\vec{V}+\nu \Delta \vec{\Omega }$$

**Figure 2:**
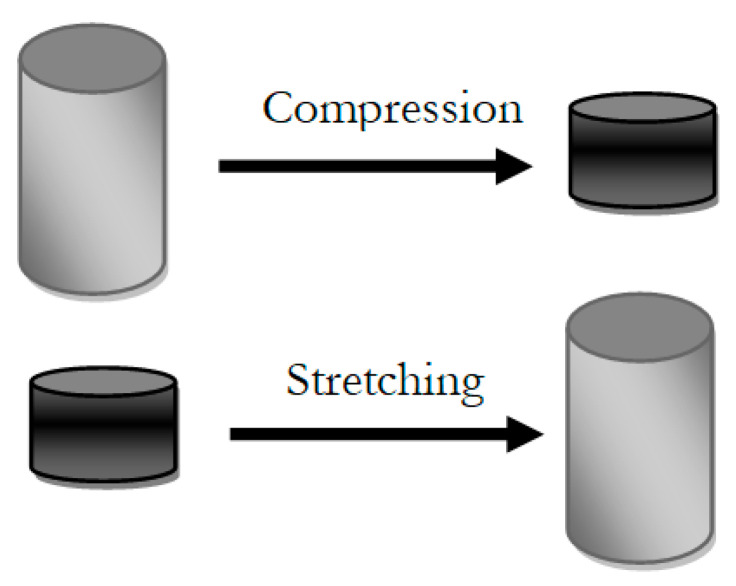
The stretching and compression processes.

The expression $\vec{\Omega }.\bar{\overset{¯}{\nabla }}\vec{V}$ provokes the development of vortex formations of different measurements by creating the compression and stretching vortex; see Figure 2. These phenomena work simultaneously on the dimensions of the vortex. At a given moment, the stretching operation develops the length of the vortex and reduces its cross-section, while the folding operation decreases the length of the vortex and augments its cross-section. These phenomena are generated as a result of the conservation of mass and kinematic moment.

Stretching leads to the divergence of neighboring points; bending leads to the mixing of distant points. These operations damage the energetic boundary layers and limit their recovery. As the boundary layer is a barrier upon parietal heat transfer, its destruction considerably improves heat and mass transfer, especially on multi-layer micromixers [18]. These processes enhance the contact surface between the fluids to be mixed, even in the presence of the interfacial barrier as a surface tension [17,18]. To define these behaviors, the coefficients of stretching and folding of the vortex α were investigated:

(7) $$A=\frac{\vec{\Omega }.\bar{\overset{¯}{D}}.\vec{\Omega }}{\Omega^{2}}$$

where$\vec{\;\Omega }$ and $\bar{\overset{¯}{D}}$ are the vorticity vector and the deformation tensor, respectively. Where *A* > 0, the vortex stretching predominates on vortex folding [19]. A− provides the division mean of the negative values of the compression parameter. A+ denotes the division average of the positive values of the stretching parameter.

### 5.2. Deformation and Vorticity Intensity

The vorticity process achieves good macroscopic homogenization, while the deformation process achieves good mixing quality. For this purpose, chaotic micromixers can be a possible solution to increase both deformation and vorticity rates. These parameters are given in the following [18,19]:

(8) $$D={\left[2{\left(\frac{\partial u}{\partial x}\right)}^{2}+2{\left(\frac{\partial v}{\partial y}\right)}^{2}+2{\left(\frac{\partial w}{\partial z}\right)}^{2}+{\left(\frac{\partial u}{\partial y}+\frac{\partial v}{\partial x}\right)}^{2}+{\left(\frac{\partial u}{\partial z}+\frac{\partial w}{\partial x}\right)}^{2}+{\left(\frac{\partial v}{\partial z}+\frac{\partial w}{\partial y}\right)}^{2}\right]}^{\frac{1}{2}}$$

(9) $$D_{\mathrm{mean}}=\frac{1}{℧}\int Sd℧$$

(10) $$\Omega =\frac{1}{2}{\left[{\left(\frac{\partial w}{\partial y}-\frac{\partial v}{\partial z}\right)}^{2}+{\left(\frac{\partial u}{\partial z}-\frac{\partial w}{\partial x}\right)}^{2}+{\left(\frac{\partial v}{\partial x}-\frac{\partial u}{\partial y}\right)}^{2}\right]}^{\frac{1}{2}}$$

(11) $$\Omega_{\mathrm{mean}}=\frac{1}{℧}\int \Omega d℧$$

where ℧ presents the total fluid volume of the micromixer.

## 6. Numerical Validation

**Figure 3:**
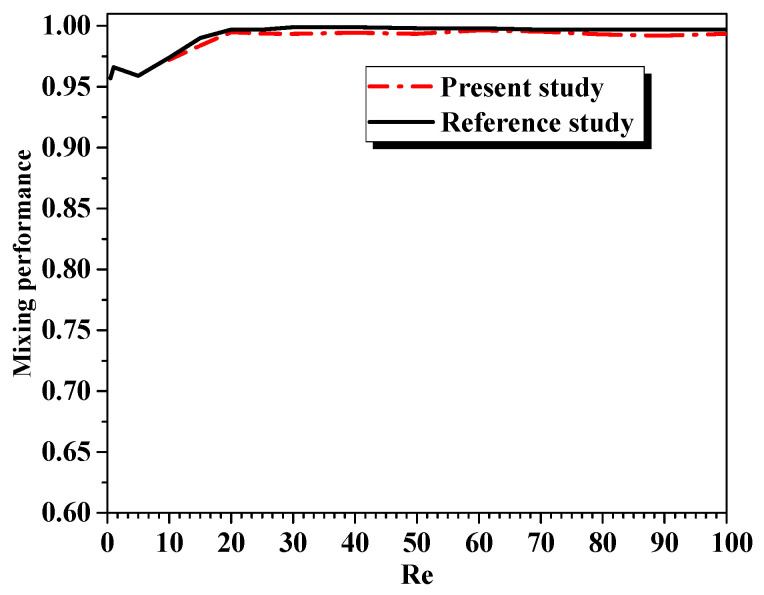
Comparison of current computational results for mixing rates at the outlet flow section with results of Jibo et al. [22] for different Reynolds numbers.

A numerical steady state of mixing flows of water fluid inside multi-layer micromixers was solved to validate the CFD accuracy with those received by Jibo et al. [22]. The results show the mixing rates as a function of various Reynolds numbers at the outlet section; see Figure 3. The computational comparison revealed satisfactory agreements among the results of Jibo et al. [22], where the relative error with the numerical results is less than 1%.

## 7. Results and Discussion

Behaviors of kinematic mixing flows for water fluid were investigated in detail for four new micromixers: TL-CM 1, TL-CM 2, TL-CM 3, and TL-CM 4, which were compared with the reference TLCCM configuration used by Kouadri et al. [11]. Local Physical Processes, such as deformation, vorticity, rotation rate, stretching and folding rates, were investigated as a function of a low Reynolds number ranging between 0.1 and 25.

**Figure 4:**
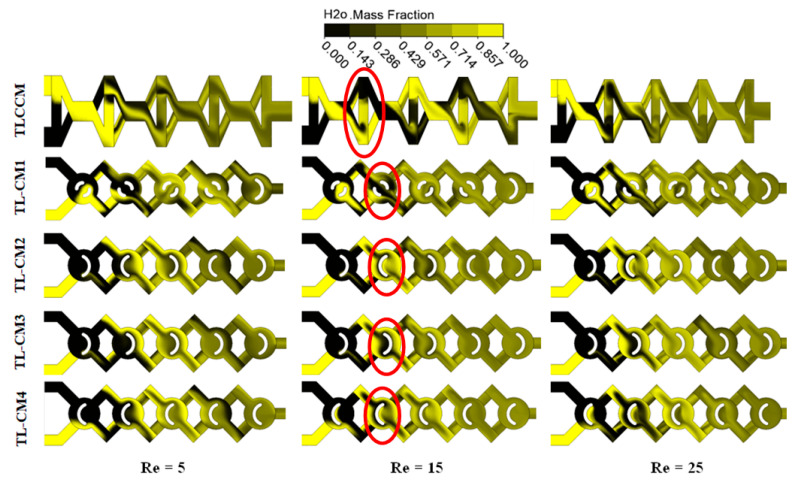
Qualitative representation of mass fraction contours for different Reynolds numbers at the horizontal middle section of each micromixer.

The flow visualization of the mass fraction between black and yellow water fluids is presented in Figure 4 for all proposed configurations. Different regimes (Re = 5, 15, and 25) were implemented within the fluid pattern to understand the development of visual mixing inside the new configuration and compared to the reference geometry. We remark that the mixing augmented with the Reynolds number for all micromixers, and there was no completely black region in the second units for all new micromixers compared to the TLCCM configuration, as can be seen in the red circle. Therefore, the new micromixers have a quicker mixing performance than TLCCM.

**Figure 5:**
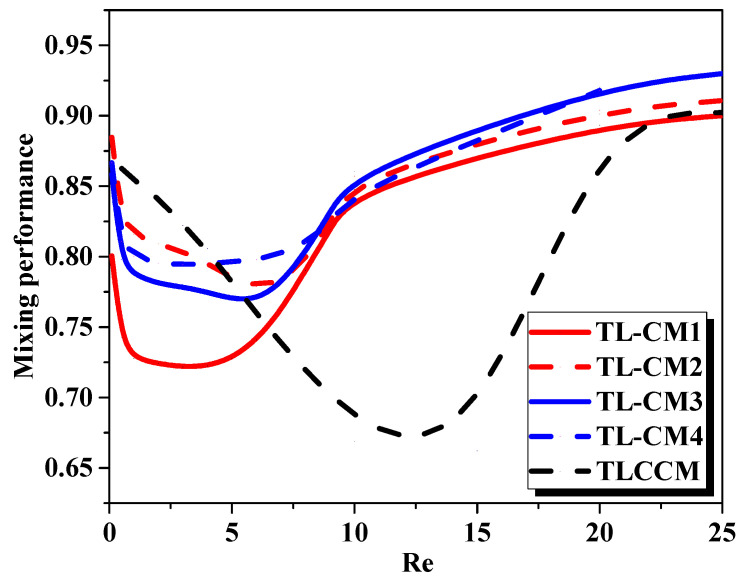
Development of mixing performance for different Reynolds numbers with various micromixers.

Figure 5 shows the evaluation of the mixing efficiency of the micromixers for various Reynolds numbers. As the flow homogenization was performed only by molecular diffusion for a very low Reynolds number (Re ≤ 5), the modified configurations were not effective in improving the mixing. When the Reynolds number increased, the fluid homogenization was more effective, and the mixing intensity developed quickly, so the most select mixing state was reached for TL-CM1, TL-CM2, TL-CM3, and TL-CM4 configurations. For the resulting plot, we found that there is scarcely a difference between the mixing rates obtained for various regimes.

**Figure 6:**
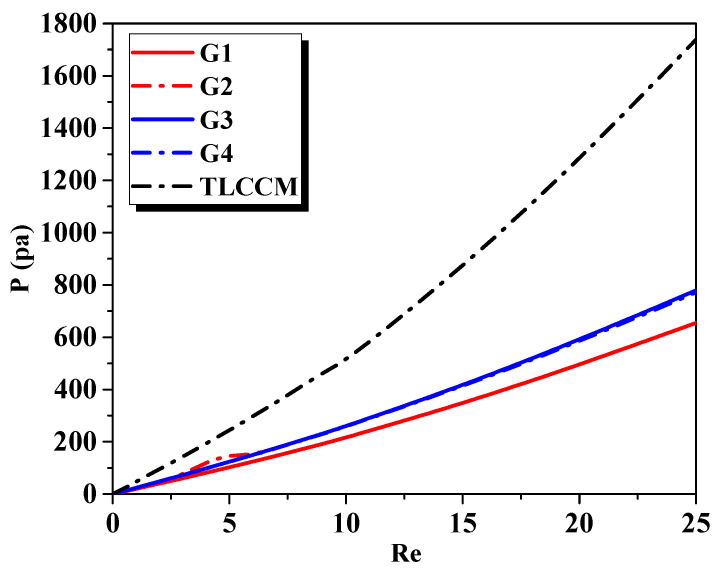
Pressure drops as a function of Reynolds number for different micromixers.

Moreover, it was observed that TL-CM4 showed a 30% enhancement of the mixing rate at the outflow with 48% lower pressure losses, as compared to the TLCCM [11] configuration at Re = 10, as shown in Figure 5 and Figure 6. The simulation demonstrated that the TL-CM4 had the greatest rates of mixing compared to the other micromixers, due to the chaotic advection impact, which confirms the fluid homogenization.

Figure 6 presents the evolutions of the pressure losses for different geometries with a low Reynolds number ranging from 0.1 to 25. The pressure tended towards asymptotic values for the new micromixer configurations. A large number of geometrical perturbations in TLLCM restricted the establishment of the boundary layer. This phenomenon increased the pressure losses, especially at TLCCM.

**Figure 7:**
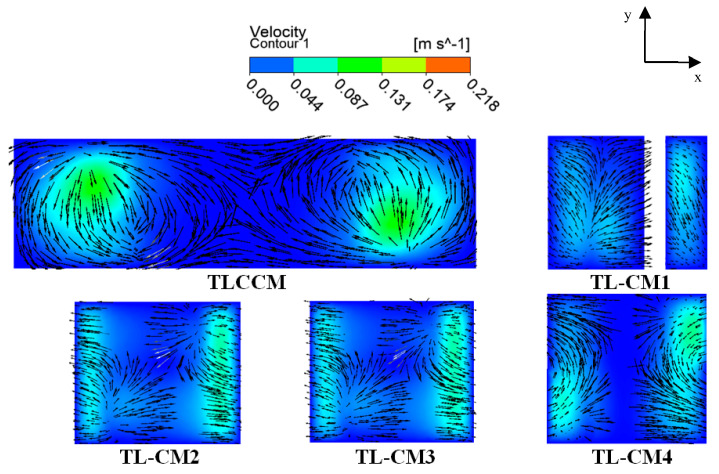
Qualitative representation of velocity vector plots and velocity contours on x-y planes of the middle third chamber with a distance of 3.25 mm from the inlet flow.

Figure 7 shows the secondary flow vectors of fluid particles at the middle regions (Re = 10) for all proposed geometries. The new configurations made a recirculation zone recorded by the sudden change in direction, due to the particular geometrical feature of the chaotic micromixer. Consequently, the main path of the vectors was distorted within 90°. Meanwhile, the vectors were observed to pass over each other, causing the fluid flow to be homogenized in the tangential direction; furthermore, significant secondary flows were created, which can contribute an extra advantage to kinematic performance and mixing fluids.

**Figure 8:**
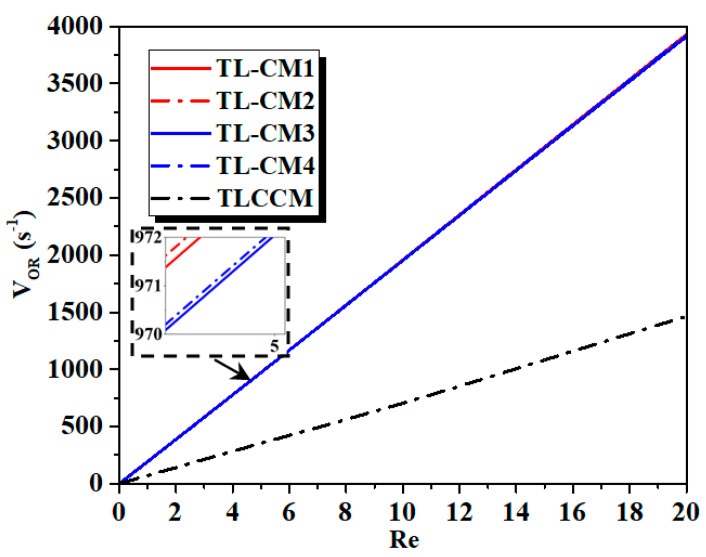
Evaluation of vortex intensity for various Reynolds numbers with different micromixers.

The effect of Reynolds numbers on the vortex intensity of the fluid within five cases of mixers is presented in Figure 8. As the Reynolds number augmented, for all micromixers, it was obvious that the flows were strong, which led to high kinematic energy. Therefore, the vorticity and secondary flow developed quickly with the Reynolds number. For a given value of Re, the dynamic flow is, likewise, powerful for the agitation of a chaotic flow within TL-CM2.

**Figure 9:**
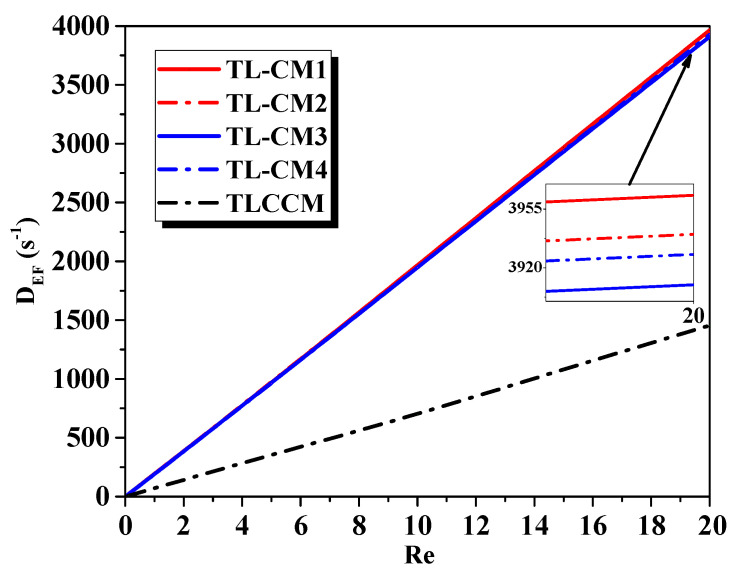
Evaluation of deformation intensity for various Reynolds numbers with different micromixers.

Equation (8) estimated the deformation intensity and the impact of the Reynolds number for different micromixers; see Figure 9. Due to the chaotic advection, the transversal movements of the particle fluid augmented, and the axial dispersion reduced the subsequently improved fluid homogenization. It was noted that the deformation rate increased with the growth of the Reynolds number. However, the permanence of the great chaotic flows in the novel shapes was compared to those revealed in the state of TLCCM. For low regimes, the evolutions were very close to each other. Consequently, the deformation rate had a lower effect on the secondary flow, and the flow passed easily within the micromixers, and thus, a vortex was not formed.

**Figure 10:**
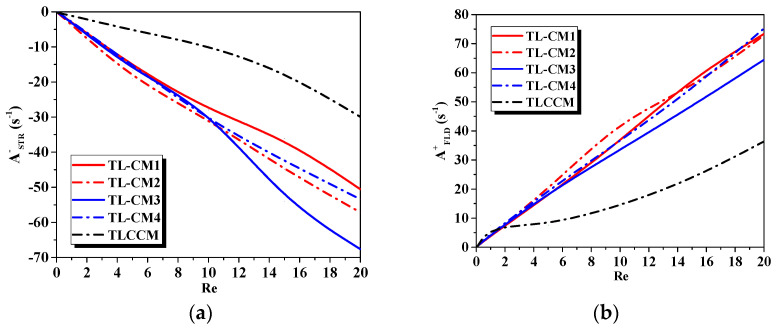
Evaluation of (**a**) stretching and (**b**) folding intensity for various Reynolds numbers with different micromixers.

Figure 10presents the developments of a compression parameter (A−) and a vortex stretching coefficient (A+) as a function of the Reynolds number for the proposed micromixers. The configurations of low Re had qualitatively the same behavior in terms of stretching and folding intensity, due to the fluid being more viscous and the secondary flows not yet being active. When the Reynolds number exceeded the value of 10, the difference enhancement was obvious for both the stretching and folding processes; as it can be seen that, when the Reynolds number increased, the intensities of the compression and stretching became larger inside the TL-CM3 and TL-CM4 geometries, respectively, compared to the others, and the fluid flow became more sheared and agitated. Moreover, the flow in TLCCM displayed low rates of stretching compared to the other micromixers.

**Figure 11:**
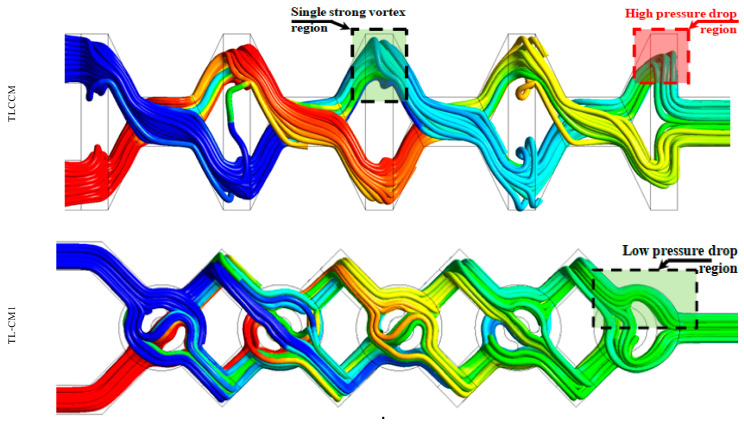

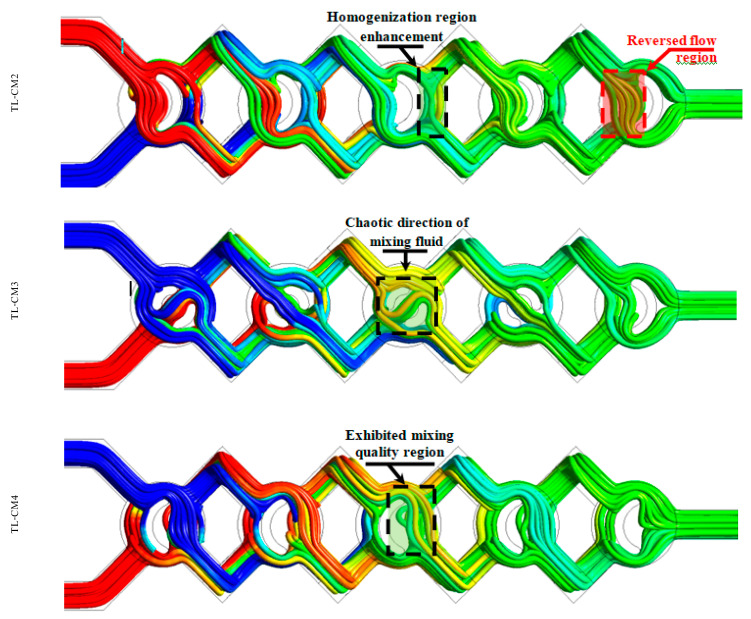
Streamlines of the mass fraction at Re = 10.

To understand the structure of the fluid flow within the micromixers, a numerical visualization of streamlines with mass fraction is presented in Figure 11. Each configuration has an important role to enhance homogenization with improved kinematic behavior. As can be seen in Figure 11, TLCCM had a single strong vortex region inside each unit which developed the mixing rate of the geometry inwardly.

However, it had a high pressure loss near the outlet part. In TL-CM2, TL-CM3, and TL-CM4 configurations, the mixing was more dynamic and chaotic because of the kinematic behavior and the existence of the intense recirculation zones in the flow, which strongly affected the hydrodynamic mixing. In addition, it was seen that the nature of the trajectories inside the selected new micromixers created a reversed flow pattern, which can enhance the kinematic characteristics and ensure high-quality mixing.

## 8. Conclusions

We examined the effective kinematic performances and homogenization fluid quality for low Reynolds numbers with four different multi-layer micromixers based on the chaotic mixing behavior. According to this work, the following remarks can be given:

- The kinematic behavior was influenced by the Reynolds number for all proposed micromixers.
- The new design contributes an enhancement advantage to kinematic measurements, especially for the folding and stretching processes.
- Strong secondary flows were created inside the new micromixer (TL-CM 3), which enhanced the mixing quality compared to the other geometries.
- As the Reynolds number increased, the flow visualization revealed that the vortex created in each micromixer has more vigorous intensity.
- TLCCM exhibited low vortex intensity of stretching and folding compared to the preferable micromixers.
- TLLCM has many geometrical perturbations and chaotic flow that increase the pressure losses within fluid flow.
- Higher rates of Reynolds number have more effects that increase both deformation and vorticity rates.
- As a consequence, for low Reynolds numbers, mixing and kinematic performances for the TL-CM 3 configuration are more important compared to the other configurations.
- The proposed micromixers might be integrated with micrototal analysis systems and LOC systems to facilitate the study of reaction kinetics, enhanced reaction selectivity, and dilution of the fluid sample.
